# Limb Salvage Versus Amputation for the Mangled Extremity: Factors Affecting Decision-Making and Outcomes

**DOI:** 10.7759/cureus.28153

**Published:** 2022-08-18

**Authors:** Isaac Okereke, Elsenosy Abdelfatah

**Affiliations:** 1 Trauma and Orthopaedics, The Royal London Hospital, London, GBR; 2 Trauma and Orthopaedics, University Hospital Dorset, Poole, GBR

**Keywords:** complex limb injuries, limb reconstruction, amputation, mangled limb, limb salvage

## Abstract

Complex limb injuries are severe injuries of the extremities that affect most or all components of the limb. Industrial/farm accidents and motor vehicle crashes are the mechanisms of injury for a large proportion of these presentations in the civilian population. While recent advances in surgery have led to more patients with complex limb injuries (that would have qualified for a primary amputation a few decades ago) undergoing limb-reconstruction surgeries, the existing evidence is inconclusive on the merits of limb salvage over amputation. Limb salvage surgery still carries considerable morbidity and mortality risks and requires careful consideration of several factors by the managing surgeons.

## Introduction and background

Complex limb injuries refer to severe injuries of the extremities that affect most anatomic components, i.e., soft tissue, osseous, neural, and vascular structures. More specifically referred to as a mangled extremity when three or more of these anatomic structures are injured [1], they result from high-energy mechanisms, can be limb-threatening as well as life-altering, and are associated with significant early and long-term morbidity. The optimal management of these complex extremity injuries requires enormous human and financial resources and is a considerable challenge to healthcare systems and the managing surgical team. There is a paucity of level I data, and the current evidence is inconclusive on the merits of limb salvage over amputation as available studies show conflicting outcomes [2].

Blunt trauma is the primary mechanism of injury for most patients who present with a complex limb injury. In civilian populations, road traffic accidents (RTA), industrial accidents, and falls are the etiology for a large proportion of these presentations. Gunshot wounds (GSW) account for approximately 2% of presentations in total [3,4]. In military personnel, the patterns and severity of injuries have evolved over the last two decades following wars in the Middle East. The recent emphasis on counterinsurgency and dismounted military operations has led to increased exposure to improvised explosive devices (IEDs) leading to injuries that have come to be known as severe dismounted complex blast injury (DCBI). DCBIs are classified as low- or high-energy injuries depending on the blast size and the distance of the victim from the explosion. High-energy injuries are characterized by injuries to both lower (usually proximal transfemoral amputations) and upper extremities (usually involving the non-dominant side), in addition to severe pelvic injuries, destructive genitourinary and abdominal trauma [5,6].

The options available for the management of a mangled extremity are either a primary amputation or an attempt at limb salvage. Attempts at salvaging a limb should only be considered if a patient is physiologically stable enough to tolerate the multiple surgical procedures required. For patients who are persistently hemodynamically unstable and those in extremis, life must come before limb. A primary amputation can be a lifesaving procedure in these circumstances for patients who are expected to return to active life. Even though the injury to a large extent determines the amputation level, the aim of surgery should be to optimize the functional outcomes of the patient by amputating as distally as possible with sufficient bone and soft tissue envelope to allow subsequent fitting of prostheses.

Limb prostheses have been in use for centuries. One of the earliest uses of a prosthesis can be seen in the mummy that is on display in the Cairo Museum, where the great toe of the right foot had been amputated and replaced with a prosthesis made out of leather and wood [7]. Limb prostheses have evolved from being just a modified crutch with a wooden or leather cup into highly sophisticated bionic limbs made of space-age materials. Complications such as non-healing wounds, skin irritation, and pain associated with socket prostheses have led to new techniques for attaching prosthetic components directly to the residual limb's skeleton, known as osseointegration. Osseointegration refers to the direct structural and functional connection between the living bone and an artificial metal implant [8,9]. Advances in bionic limbs have led to osseointegration of limb prosthesis, the development and wide adoption of implanted muscle electrodes for decoding, and implanted nerve electrodes for sensory feedback [10]. Unfortunately, upper limb prostheses have not recorded as much success as lower limb prostheses, and currently available ones only allow for basic movements.

## Review

Clinical assessment

The assessment of a patient with a complex limb injury poses a significant challenge for the surgical teams. These patients require rapid and timely evaluation for prompt identification of injuries that may either require a primary amputation or potentially benefit from salvage attempts.

Patients with a mangled extremity presenting to the emergency department should be trauma-called and ideally should trigger the massive transfusion protocol (MTP). The initial clinical evaluation should be a rapid assessment of the ABCs (airway, breathing, and circulation) through a systematic primary survey following the advanced trauma life support (ATLS) protocol. The British Orthopaedic Association's Standards for Trauma (BOAST) guidelines recommend early antibiotic therapy in patients with an open fracture, ideally within one hour of injury [11].

During the clinical assessment, the presence of injuries such as supracondylar femoral fractures, knee dislocations, high-energy proximal tibia fracture patterns (Schatzker type IV and above), and penetrating injuries of the medial and posterior compartments of the thigh should prompt a high index of suspicion of an associated vascular injury. Also, the presence of "hard" or "soft" vascular signs can be of significant utility in the evaluation of an injured extremity. Frykberg in 1992 described hard vascular signs as the absence of a distal pulse, distal ischemia, ongoing hemorrhage, a large hematoma, bruit, or a thrill on palpation. They further described soft signs as a history of hemorrhage, the presence of a small hematoma, hypotension, and an associated nerve deficit [12]. There is a controversy on the effectiveness of making clinical decisions on the basis of the presence of these signs. In their study published in 2021, Romagnoli et al. suggested that the hard signs of vascular injury have significant limitations in identifying and characterizing extremity arterial injuries [13].** **Undertaking a computed tomography angiography (CTA) of the vessel in question is now the gold standard in evaluating patients with a potential vascular injury. Current treatment guidelines recommend emergent surgical exploration with imaging beyond routine plain films contraindicated for patients with "hard" signs of arterial injury [14]. Also, patients presenting with no hard vascular signs and abnormal physiologic parameters (with or without an ankle-brachial index [ABI] measurement < 0.9) should have further imaging to rule out an arterial injury.

Achieving hemostasis early on is essential during the initial management of patients with a mangled extremity. The presence of catastrophic hemorrhage with hemodynamic instability from a mangled extremity implies that bleeding control takes precedence over the ABCs of the ATLS protocol [15]. In this acute phase, damage control orthopedics (DCO) with temporizing strategies such as the use of an external fixation device, multicompartment fasciotomies, and temporary vascular shunting, implemented concurrently with damage control resuscitation measures, have been shown to be effective. Hemostasis can be achieved initially by the direct application of pressure over the bleeding vessel in most cases. Furthermore, the use of pneumatic tourniquets for achieving proximal control of extremity bleeding in the pre-hospital and emergency setting is recommended and routinely employed as they have low rates of complications in contrary to the misconceptions about their predisposition to cause compartment syndromes [16]. Hemorrhages from sources that are not amenable to tourniquet control may benefit from the application of hemostatic dressings such as HemCon® (HemCon Medical Technologies, Inc., Portland, Oregon) and QuickClot® (Henry Schein, Inc., Melville, New York), while bleeding from areas that are difficult to access or from non-compressible regions like the groin, axilla, or deep thigh may require an extraluminal balloon tamponade to control bleeding and allow for careful wound exploration. Intraluminal occlusion balloons are also a useful temporary adjunct utilized in damage control surgery to control back-bleeding from distal vascular segments allowing for a clear field for repair to be attempted [17]. When salvage is considered for a mangled extremity, an intraluminal shunt can be inserted temporarily to perfuse the limb's distal segment. The "Belfast approach" recommends early placement of shunts in both artery and vein with a plan to reconstruct all anatomical structures in the first encounter [18]. Warm ischaemia time should ideally not exceed six hours in the lower extremity and eight hours in the upper extremity [4].

Following temporary or definitive bleeding control and stabilization of the physiologic status, patients with a mangled limb can have their wounds explored at this stage, and any associated fractures or joint dislocations should have basic plain films in two orthogonal views. First, dislocated joints should be carefully relocated with the application of temporary external fixations of fractures and dual-incision fasciotomies where necessary, and decisions for a complex reconstruction or straightforward repair for the vascular injuries, where present, were made.

The patient's baseline health status contributes significantly to determining what management course is opted for [19]. In a level IV study, de Mestral et al. found that patients who underwent early amputations were on average older than those who had limb salvage. Also, associated factors such as involvement in a road traffic collision (RTC), the presence of shock in ED, an associated head injury, and crush injury around the popliteal region are correlated with a higher frequency of early amputation [20].

To salvage or amputate?

Most mangled limbs can be salvaged after trauma with advances in skeletal fixation, limb reconstruction, wound care, microsurgery, and autologous vascularized tissue transfer. Thus, the dilemma that reconstructive surgeons face is no longer how but whether attempting salvage is the best treatment option for the patient.

The decision to salvage or amputate a limb is challenging for the managing team as they are faced with several factors that require thoughtful consideration before embarking on either the salvage surgery's long and winding path or the irreversible and final decision to amputate a limb. Important variables for contemplation include, but are not limited to, the patient's age and pre-injury state of health; the level of proposed amputation determined by the degree and severity of damage to the extremity; the patient's physical, psychological, and socioeconomic status; and available support system [21].

Several clinical scores and guidelines have been developed to aid clinical decision-making in order to minimize the morbidity and mortality associated with a failed limb salvage attempt [22]. The Gustilo-Anderson classification of open fractures (see Table 1) was first reported in 1976 and classified open fractures into types I to III, and was further revised in 1984 to subclassify type III injuries. It is a primarily descriptive system developed to stratify the risk of infections in open lower extremity fractures only [23]. Injuries classified as Gustilo-Anderson type III, the most severe injuries, are associated with a 50% primary amputation rate [24]. In their work, Caudle and Stern showed the prognostic relevance of Gustilo-Anderson subclassification of type III injuries in determining infection, non-union, and amputation rates [25]. Owing to the high interobserver variability of type III injuries and discrepant reports of amputation within its different subclasses, attempts were made to develop classification systems that are more predictive and holistic while giving due consideration to other determinants of outcomes such as the patient's demographics, hemodynamic stability, and the presence of nerve injuries.

**Table 1:** The modified Gustilo-Anderson classification of open fractures

Type	Description
I	A fracture with a clean laceration < 1 cm in length, caused by low-velocity trauma and minimal contamination
II	A fracture with a laceration > 1 cm in length lacking any severe soft tissue damage or devitalized tissue
III	A fracture with extensive soft-tissue loss
IIIA	Adequate coverage of fracture by soft tissue despite extensive cutaneous laceration or flaps, or high-energy trauma regardless of the size of the wound
IIIB	More extensive injury and contamination of the soft tissue, periosteal stripping, and soft-tissue gaps present with exposed bone; will likely require either a local soft-tissue flap or a free flap for coverage
IIIC	Any open fracture with an arterial injury requires repair regardless of the degree of soft-tissue disruption

The mangled extremity severity score (MESS), designed by Johansen et al. [26], is a simple rating scale for lower extremity trauma based on four significant criteria: patient's age, limb ischemia, shock, and skeletal or soft-tissue damage (see Table 2).

**Table 2:** Mangled extremity severity score (MESS) *Score doubled for ischemia > 6 hours. MVA: Motor vehicle accidents; GSW: Gunshot wounds.

A. Skeletal/soft-tissue injury
1. Low energy (stab; simple fracture; pistol gunshot wound)
2. Medium energy (open or multiple fractures, dislocation)
3. High energy (high-speed MVA or rifle GSW)
4. Very high energy (high-speed trauma + gross contamination)
B. Limb ischemia
1. Pulse reduced or absent but perfusion normal*
2. Pulseless; paresthesia, diminished capillary refill
3. Cool, paralyzed, insensate, numb*
C. Shock
0. Systolic BP always > 90 mmHg
1. Hypotensive transiently
2. Persistent hypotension
D. Age (years)
0. <30
1. 30-50
2. >50

The investigators retrospectively examined 25 patients with severe limb injuries for objective factors that may predict amputation and found a significant difference in the mean MESS between amputated and salvaged limbs (4.88 vs 9). Patients with a sciatic or posterior tibial nerve disruption were excluded from this study. The MESS aids clinical decision-making by objectively determining complex limb injuries that warrant a primary amputation [26]. Helfet et al. found MESS scores > 7 to have a positive predictive value for amputation [27]. MESS scores are doubled for a warm ischemia time above six hours, and this is an absolute contraindication for attempting limb salvage [28]. Other less frequently employed scores include the predictive salvage index (PSI), the mangled extremity syndrome index (MESI), the Hannover Fracture Scale, the nerve injury, ischemia, soft tissue, skeletal injury, shock, age of patient score (NISSSA), and the limb salvage index (LSI) [28,29]. These scoring indices attempt to use numerical scores to prognosticate whether to attempt salvage or primarily amputate an injured limb. The disadvantage of these systems is that they are quite cumbersome to establish in the emergency setting as required details on some scoring domains cannot be entirely gleaned in the emergency department. Also, the attempt to assign numerical values to objective data leads to considerable interobserver variability and, consequently, poor reliability [30]. In essence, scoring systems have limited usefulness and should not be used as the sole basis for the decision to amputate is made.

The Lower Extremity Assessment Project (LEAP) was a multicenter, prospective, observational study done to determine the functional outcomes of 569 patients with severe leg injuries that resulted in either limb salvage or amputation. The Sickness Impact Profile (SIP), a multidimensional measure of self-reported health status, was the principal outcome measure [31]. While this study concluded that soft-tissue injury severity had the most significant impact on whether to proceed with limb salvage or amputation, it also showed the variability of what was considered the most critical factor in deciding to salvage a limb, further emphasizing the need for individually tailored treatment for patients. Indications for early amputation include the presence of bony or soft-tissue injuries that are not amenable to reconstruction, vascular injuries that are irreparable, severe loss of skin and soft tissues in the plantar area of the foot, near-complete amputation, massive crush injuries, extensive neurovascular damage, and consensus at multidisciplinary team (MDT) meeting.

A multidisciplinary team approach that considers all the variables previously mentioned in their decision-making process is essential for achieving the best outcomes for each case.

Outcomes following limb salvage versus amputation

The Patient Experience Questionnaire PEQ, SF-36, and the SIP are the three widely validated instruments for outcome measures in patients who have suffered extremity trauma [32-34]. Hoogendoorn and van der Werken reviewed the quality of life (QOL) and postoperative functional outcomes in amputees compared to patients with successful limb salvage surgery who had suffered a Gustilo-Anderson grade III open tibial fracture and concluded that the successful salvage surgery cohort experienced considerably more complications requiring further surgical interventions than those who underwent primary amputation [32]. Conversely, MacKenzie et al. found poor functional outcomes with significant psychosocial and physical disability in patients with lower extremity amputation following trauma at 24 months [33]. Additionally, Bosse et al., in a multicenter, prospective, observational study, found no significant difference in the SIP scores between patients who had an amputation or limb salvage surgery (12.6 vs 11.8, P = 0.53). This study determined the predictors of a more inferior SIP score to include significant complications requiring rehospitalization, low educational level, non-White race, low socioeconomic status, a weak or absent social-support network, low self-efficacy, and smoking [34].

Similarly, in a meta-analysis of relevant studies, Akula et al. demonstrated that limb salvage surgery in patients with mangled extremities had better outcomes psychologically for the patients than amputees; however, there was no difference in terms of physical outcomes between the two groups [35]. While these results have been reinforced by the LEAP study, which found no difference in the functional outcomes between patients who underwent limb salvage or amputation at two- and seven-year follow-ups [36], the Military Extremity Trauma Amputation/Limb Salvage (METALS) study reported different results. The METALS study was a retrospective cohort study of 324 military personnel who sustained a lower limb injury treated either with an amputation or limb salvage. This study found amputees to not only have better functional scores but was able to carry out more intense physical activity compared to those treated with limb salvage [37,38]. The authors conjectured that the reason for the difference in outcomes compared to the LEAP study was that military amputees received more focused rehabilitation early on in their recovery [38].

## Conclusions

The primary goal of treating a mangled extremity is to achieve optimal limb function and avoid unnecessary interventions where possible. Although the number of patients undergoing limb reconstruction surgeries has increased exponentially in the last decade due to advancements in reconstructive techniques, limb salvage surgery still carries considerable morbidity and mortality risks. On the other hand, early amputation affords patients a quicker recovery time, shorter hospital stays, and reduced financial implications. Amputations are, however, life-changing procedures that require significant functional adjustments for the patient. Therefore, a holistic methodology that considers these variables by employing a multidisciplinary approach when deciding surgical management options for patients with complex limb injuries is imperative. The patient must be carried along in the clinical decision-making process from the outset, and their wishes must be always respected by the managing team.

In conclusion, it is worth noting that a functional extremity, for instance, might be a below-knee amputation with an early prosthetic fitting as such a patient with that degree of recovery following a severe limb injury would most likely report better functional outcomes than a person with a dysfunctional limb in whom salvage has been attempted. Furthermore, while a limb amputation may give better early functional results at reduced costs, when assessed over the course of the patient's life, limb salvage may provide improved function at a lower cost overall.
